# Supplementary material for: The Genome Assembly and Annotation of the Southern Elephant Seal Mirounga leonina
Source: Genes (Basel). 2020 Feb 3;11(2):160. doi: 10.3390/genes11020160 (PMC7073746; doi:10.3390/genes11020160)
Supplement: Supplementary file 1 [file genes-11-00160-s001.zip › Supplementary Figure S1-S4.pdf]

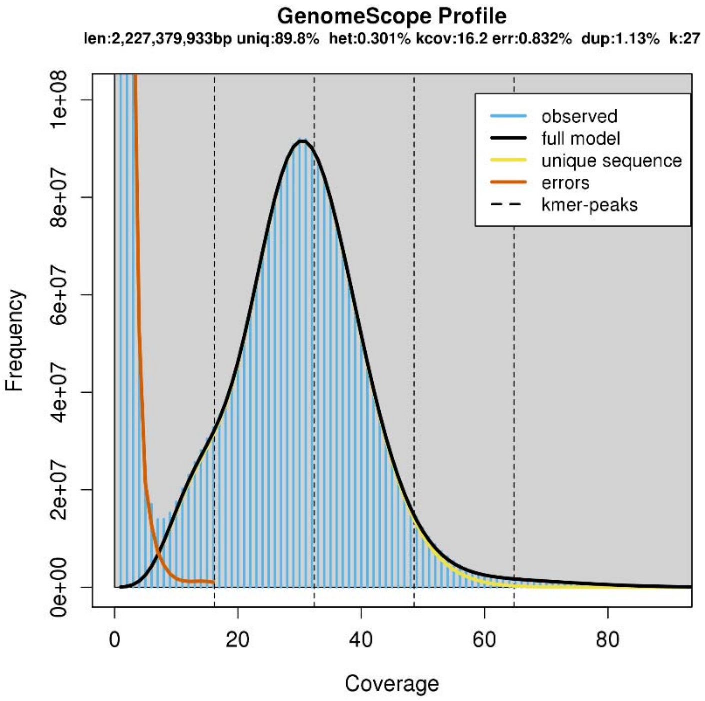

Supplementary Figure S1. Graph of the k-mer distribution (K = 27) using GenomeScope (http://qb.cshl.edu/genomescope/).

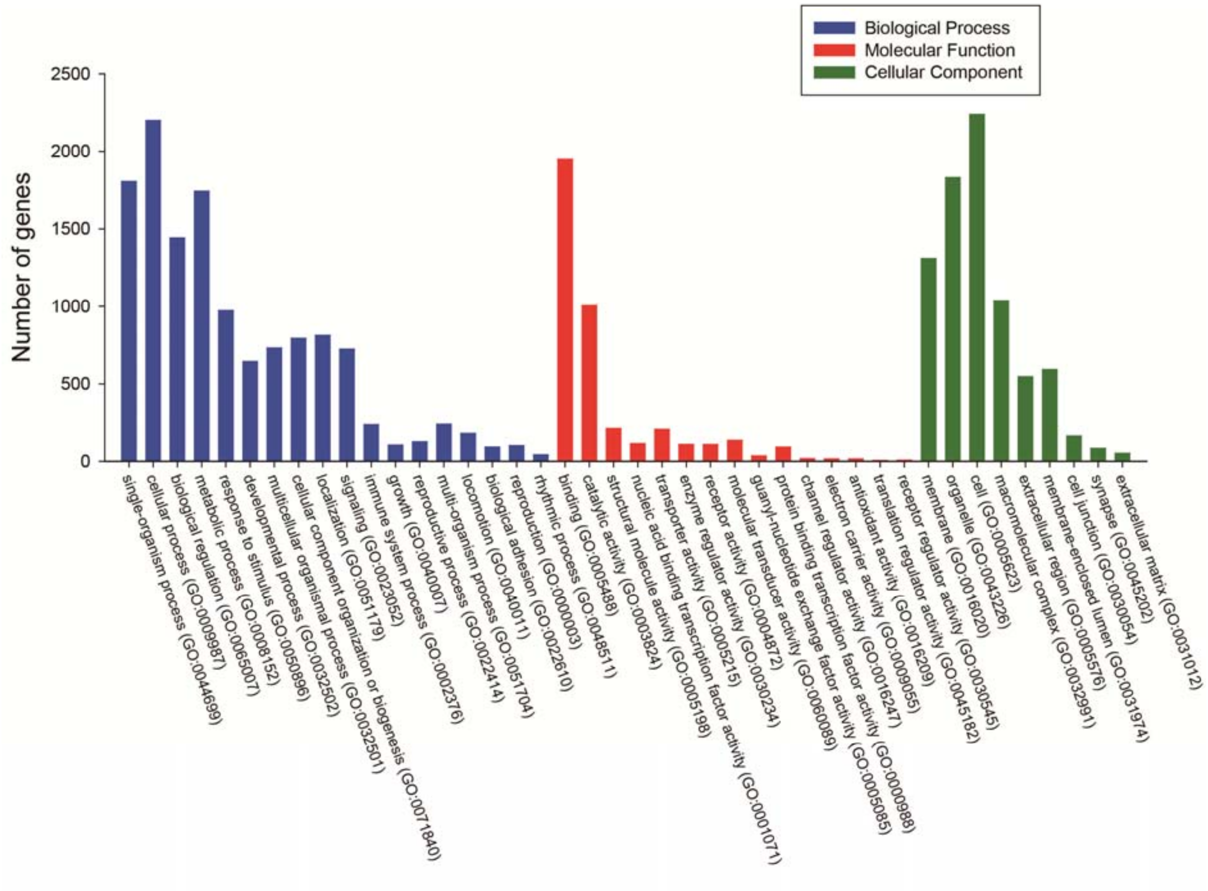

Supplementary Figure S2. Gene ontology (GO), The horizontal axis indicates classes of the second-level GO-annotation, and the vertical axis indicates the number of genes in each class.

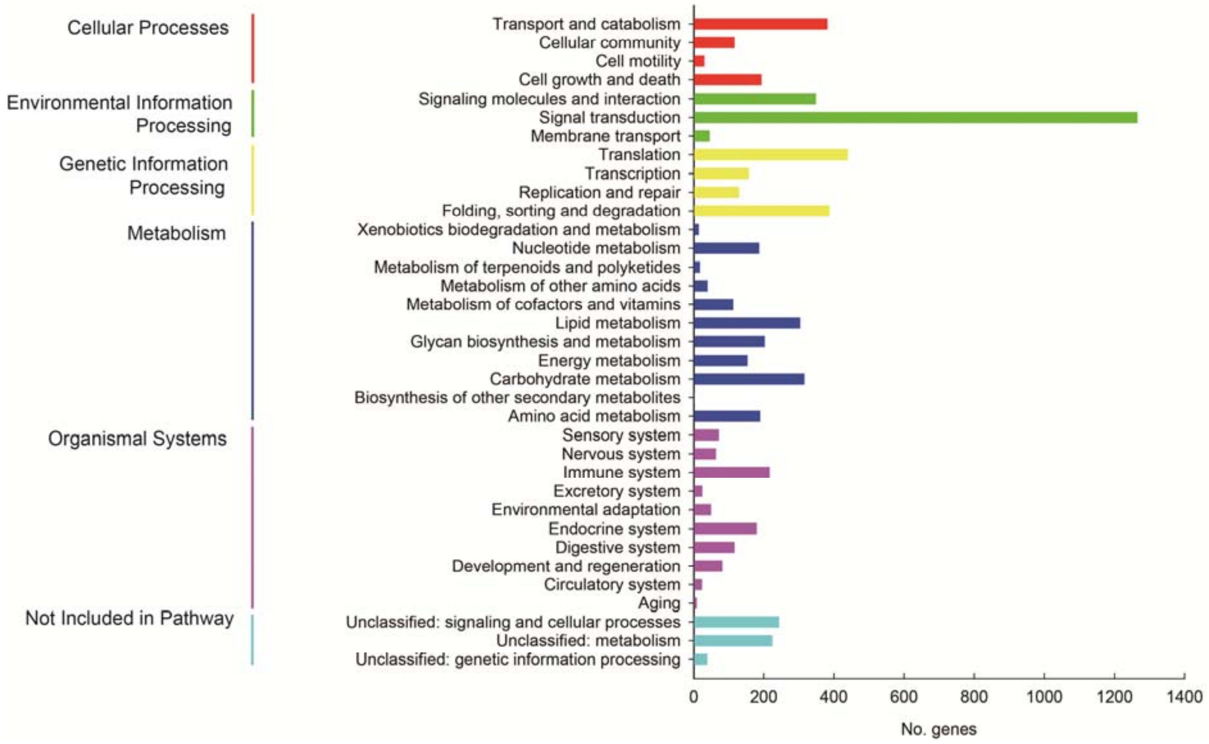

Supplementary Figure S3. Statistics of Kyoto Encyclopedia of Genes and Genomes (KEGG) classifications.

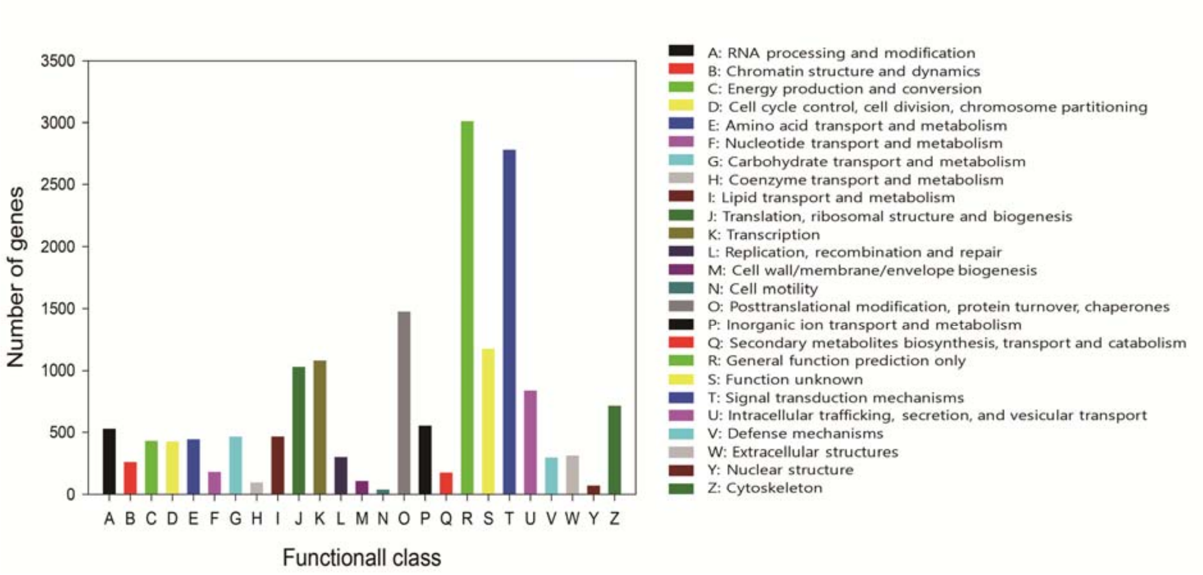

Supplementary Figure S4. Eukaryotic Orthologous Groups (KOG) classification of the predicted genes. Results are grouped into 24 functional classes according to their functions. The horizontal axis indicates each class, and the vertical axis indicates the number of genes in each class.
