# Supplementary material for: The Genome Assembly and Annotation of the Southern Elephant Seal Mirounga leonina
Source: Genes (Basel). 2020 Feb 3;11(2):160. doi: 10.3390/genes11020160 (PMC7073746; doi:10.3390/genes11020160)
Supplement: Supplementary file 1 [file genes-11-00160-s001.zip › Supplementary Table S1.pdf]

Supplementary Table S1. Lengths of Southern elephant seal genome assembly (over 1Mb).

No.	Scaffold name	length (bp)
1	Mir_151	111,625,095
2	Mir_115	106,266,018
3	Mir_53	104,114,609
4	Mir_173	94,035,216
5	Mir_174	84,269,387
6	Mir_165	82,716,099
7	Mir_129	81,593,014
8	Mir_125	80,451,303
9	Mir_127	80,439,415
10	Mir_168	75,921,071
11	Mir_154	65,650,358
12	Mir_176	60,168,797
13	Mir_82	59,607,343
14	Mir_94	58,924,836
15	Mir_93	57,187,535
16	Mir_114	54,232,831
17	Mir_91	50,706,163
18	Mir_170	46,862,779
19	Mir_153	43,798,732
20	Mir_128	41,632,241
21	Mir_166	38,232,912
22	Mir_150	37,459,734
23	Mir_180	37,025,656
24	Mir_175	36,626,112
25	Mir_126	35,720,745
26	Mir_201	32,483,541
27	Mir_418	31,866,211
28	Mir_101664	30,109,608
29	Mir_200	29,771,347
30	Mir_178	29,049,964
31	Mir_366	29,006,657
32	Mir_92	26,897,518
33	Mir_95	25,870,752
34	Mir_204	24,646,407
35	Mir_171	24,488,919
36	Mir_104	23,388,999
37	Mir_169	23,083,835
38	Mir_152	21,617,366
39	Mir_224	21,430,493
40	Mir_454	20,220,240
41	Mir_101595	19,151,837

42	Mir_357	17,326,455
43	Mir_172	16,954,670
44	Mir_197	15,716,247
45	Mir_101609	15,202,017
46	Mir_447	15,156,327
47	Mir_199	14,721,163
48	Mir_195	12,832,078
49	Mir_488	12,746,847
50	Mir_54	12,384,050
51	Mir_376	11,830,400
52	Mir_55	11,635,964
53	Mir_177	10,260,853
54	Mir_268	10,036,466
55	Mir_97	9,444,568
56	Mir_96	9,442,007
57	Mir_99	8,611,262
58	Mir_98	8,609,874
59	Mir_179	8,010,380
60	Mir_118	6,720,588
61	Mir_117	6,719,996
62	Mir_106	6,504,906
63	Mir_107	6,496,045
64	Mir_507	6,050,203
65	Mir_101608	6,002,376
66	Mir_108	5,736,955
67	Mir_109	5,734,073
68	Mir_321	5,245,318
69	Mir_101799	5,037,312
70	Mir_260	4,546,176
71	Mir_493	4,224,979
72	Mir_413	4,158,172
73	Mir_167	4,125,719
74	Mir_101414	3,788,321
75	Mir_101529	3,659,992
76	Mir_353	3,641,666
77	Mir_205	3,627,101
78	Mir_481	3,604,263
79	Mir_239	3,467,125
80	Mir_448	3,325,889
81	Mir_83	3,233,538
82	Mir_84	3,230,480
83	Mir_101283	3,113,632
84	Mir_375	2,955,563

85	Mir_242	2,875,773
86	Mir_101222	2,191,501
87	Mir_347	1,489,598
88	Mir_364	1,366,564
89	Mir_101097	1,356,989
90	Mir_363	1,255,130
91	Mir_228	1,231,732
92	Mir_234	1,168,029
