# Supplementary material for: The Genome Assembly and Annotation of the Southern Elephant Seal Mirounga leonina
Source: Genes (Basel). 2020 Feb 3;11(2):160. doi: 10.3390/genes11020160 (PMC7073746; doi:10.3390/genes11020160)
Supplement: Supplementary file 1 [file genes-11-00160-s001.zip › Supplementary Table S2.pdf]

Supplementary Table S2. Completeness of the southern elephant seal genome assembly evaluated with benchmarking universal single-copy orthologs (BUSCO).

**vertebrata_odb9**	**No**	**%**
Complete BUSCOs (C)	2,472	95.6
Complete and single-copy BUSCOs (S)	2,375	91.8
Complete and duplicated BUSCOs (D)	97	3.8
Fragmented BUSCOs (F)	65	2.5
Missing BUSCOs (M)	49	1.9
Total BUSCO groups searched	2,586	
