# Supplementary material for: The Genome Assembly and Annotation of the Southern Elephant Seal Mirounga leonina
Source: Genes (Basel). 2020 Feb 3;11(2):160. doi: 10.3390/genes11020160 (PMC7073746; doi:10.3390/genes11020160)
Supplement: Supplementary file 1 [file genes-11-00160-s001.zip › Supplementary Table S3.pdf]

Supplementary Table S3. Mapping of southern elephant seal genome assembly to Califonia sea lion chromosomes (assembly zalCal2.2).

| Cat (*Felis catus*) chromosome | chromosome length (bp) | Southern elephant seal scaffolds | scaffolds length (bp) |
|---|---|---|---|
| NW_020884868 | 212,595,508 | Mir_115 | 106,266,018 |
| | | Mir_94 | 58,924,836 |
| | | Mir_169 | 23,083,835 |
| | | Mir_172 | 16,954,670 |
| NW_020874464 | 200,785,782 | Mir_129 | 81,593,014 |
| | | Mir_176 | 60,168,797 |
| | | Mir_128 | 41,632,241 |
| | | Mir_357 | 17,326,455 |
| NW_020884869 | 192,866,806 | Mir_151 | 111,625,095 |
| | | Mir_127 | 80,439,415 |
| NW_020874458 | 187,371,479 | Mir_173 | 94,035,216 |
| | | Mir_165 | 82,716,099 |
| NW_020874474 | 149,267,531 | Mir_168 | 75,921,071 |
| | | Mir_95 | 25,870,752 |
| | | Mir_104 | 23,388,999 |
| NW_020874467 | 145,027,273 | Mir_53 | 104,114,609 |
| | | Mir_366 | 29,006,657 |
| | | Mir_54 | 12,384,050 |
| | | Mir_55 | 11,635,964 |
| NW_020876041 | 143,424,588 | Mir_82 | 59,607,343 |
| | | Mir_91 | 50,706,163 |
| | | Mir_178 | 29,049,964 |
| NW_020875357 | 138,136,661 | Mir_125 | 80,451,303 |
| | | Mir_150 | 37,459,734 |
| | | Mir_195 | 12,832,078 |
| NW_020884870 | 137,106,722 | Mir_175 | 36,626,112 |
| | | Mir_126 | 35,720,745 |
| | | Mir_201 | 32,483,541 |
| | | Mir_418 | 31,866,211 |
| NW_020874514 | 124,361,606 | Mir_101664 | 30,109,608 |
| | | Mir_204 | 24,646,407 |
| | | Mir_101595 | 19,151,837 |
| | | Mir_101609 | 15,202,017 |
| | | Mir_268 | 10,036,466 |
| NW_020879070 | 111,938,561 | Mir_153 | 43,798,732 |
| | | Mir_166 | 38,232,912 |
| | | Mir_92 | 26,897,518 |
| NW_020874544 | 109,062,339 | Mir_154 | 65,650,358 |
| | | Mir_175 | 36,626,112 |

| | | | |
|---|---|---|---|
| | | Mir_152 | 21,617,366 |
| | | Mir_454 | 20,220,240 |
| NW_020876465 | 105,284,314 | Mir_174 | 84,269,387 |
| | | Mir_199 | 14,721,163 |
| NW_020874788 | 92,542,987 | Mir_170 | 46,862,779 |
| | | Mir_224 | 21,430,493 |
| | | Mir_447 | 15,156,327 |
| NW_020878390 | 90,610,359 | Mir_93 | 57,187,535 |
| | | Mir_171 | 24,488,919 |
| NW_020874462 | 86,896,367 | Mir_114 | 54,232,831 |
| | | Mir_152 | 21,617,366 |
| NW_020875730 | 59,709,859 | Mir_200 | 29,771,347 |
| | | Mir_197 | 15,716,247 |
| | | Mir_488 | 12,746,847 |
| NW_020874921 | 59,282,897 | Mir_180 | 37,025,656 |
| | | Mir_376 | 11,830,400 |
| | | Mir_177 | 10,260,853 |
